# A Novel Photocatalyst with Ferromagnetic Core Used for the Treatment of Olive Oil Mill Effluents from Two-Phase Production Process

**DOI:** 10.1155/2013/196470

**Published:** 2013-12-31

**Authors:** Javier Miguel Ochando-Pulido, Gassan Hodaifa, María Dolores Víctor-Ortega, Antonio Martínez-Ferez

**Affiliations:** ^1^Chemical Engineering Department, University of Granada, 18071 Granada, Spain; ^2^Molecular Biology and Biochemical Engineering Department, University Pablo de Olavide, 14013 Seville, Spain

## Abstract

Photocatalytic degradation of olive oil mill wastewater from two-phase continuous centrifugation process was studied. A novel photocatalyst with ferromagnetic properties was characterized and investigated. The degradation capacity of the photocatalytic process of olive oil washing wastewater (OMW) and mixture of olives and olive oil (1 v/v) washing wastewaters (MOMW) was demonstrated. At lab-scale, the %COD removal and residence time (*τ*) for MOMW and OMW were 58.4% (*τ* = 2 h) and 21.4% (*τ* = 3 h), respectively. On the other hand, at pilot scale, 23.4% COD_removal_, 19.2% total phenols_removal_, and 28.1% total suspended solids_removal_ were registered at the end of the UV/TiO_2_ process for OMW, whereas 58.3% COD_removal_, 27.5% total phenols_removal_, and 25.0% total suspended solids_removal_ for MOMW. Also, before the UV/TiO_2_ reaction, a pH-T flocculation operation as pretreatment was realized. The overall efficiency of the treatment process for MOMW was up to 91% of COD_removal_, in contrast with 33.2% of COD_removal_ for OMW.

## 1. Introduction

**Figure 1:**
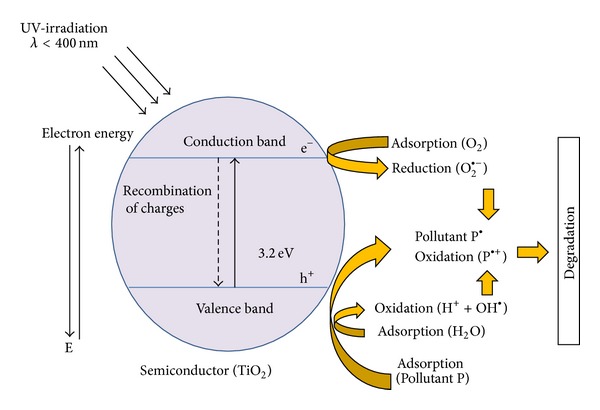
Activation of the TiO_2_ nanocatalyst by UV light.

Photocatalysis with titanium dioxide (TiO_2_) under ultraviolet light (UV) is an advanced oxidation process (AOP) in which the titanium dioxide semiconductor absorbs UV radiation and generates hydroxyl radicals (OH^●^). In detail, conduction band electrons and valence band holes of TiO_2_ are initially yielded by UV irradiation. Thereafter, band electrons are capable of interacting with surface-adsorbed molecular oxygen to form superoxide radical anions (O_2_^●^), whereas band holes will interact with water to produce hydroxyl radicals (OH^●^) (Figure 1) [1]. Organic compounds can undergo oxidative degradation through their reactions with valence band holes, hydroxyl (OH^●^), and superoxide (O_2_^●^) radicals as well as reductive cleavage through their reactions with electrons (e^−^).

The titanium dioxide molecule absorbs near UV radiation (wavelength *λ* < 400 nm), leading to the generation of electron/valence band holes pairs, as indicated as follows (Figure 1):

(1) $$\begin{array}{c} {\text{TiO}}_{2}\left(h\cdot v\right)\to {\text{TiO}}_{2}\left({\text{e}}^{-}+{\text{h}}^{+}\right) \end{array}$$

Valence band holes are prone to react with absorbed substances, in particular with water molecules (H_2_O_ad_) (2) or hydroxyl ions (OH^−^_ad_) (3), generating hydroxyl radicals (OH^●^) as follows:

(2) $$\begin{array}{c} {\text{TiO}}_{2}\left({\text{h}}^{+}\right)+{\text{H}}_{2}{\text{O}}_{\text{ad}}\to {\text{TiO}}_{2}+{\text{OH}}^{●}+{\text{H}}^{+} \end{array}$$

(3) $$\begin{array}{c} {\text{TiO}}_{2}\left({\text{h}}^{+}\right)+{{\text{OH}}^{-}}_{\text{ad}}\to {\text{TiO}}_{2}+{\text{OH}}^{●} \end{array}$$

Absorbed oxygen is the main electron acceptor species as follows:

(4) $$\begin{array}{c} {\text{TiO}}_{2}\left({\text{e}}^{-}\right)+{\text{O}}_{2}\to {\text{TiO}}_{2}+{{\text{O}}_{2}}^{●} \end{array}$$

Among the known semiconductor photocatalysts (ZnO, WO_3_, CdS, ZnS, SrTiO_3_, SnO_2_, Fe_2_O_3_) TiO_2_ has deserved particular interest owed to its high oxidizing power of organic contaminants along with its chemical stability and low cost [2]. The key advantages of TiO_2_/UV light photocatalysis process rely on the very high volume to surface ratio of titania nanoparticles that lead to the absence of mass transfer limitations when nanoparticles are used as photocatalysts, the possibility of being triggered at ambient operating conditions and to exploit sunlight instead of electric illumination in case of doped TiO_2_, and the fact that TiO_2_ is a highly active and nontoxic molecule capable of achieving complete mineralization of a wide range of organic pollutants or oxidizing them into harmless compounds [3, 4].

Olive oil factories, commonly known as olive mills, generate as by-product effluents (OME) an average daily amount of 1 m^3^ of wastewater derived from the washing of the olives (olives washing wastewater, OWW) together with more than 10 m^3^ of wastewater coming from the centrifugation process used for the extraction of the olive oil (olive oil mill wastewater, OMW). OMW exhibits a series of characteristics that make their reclamation by conventional physicochemical treatments extremely difficult. The presence of phytotoxic refractory pollutants, such as phenolic compounds, organic acids, tannins, long chain fatty acids, and organohalogenated contaminants, makes these effluents recalcitrant to biological degradation and thus inhibits the efficiency of biological processes. Moreover, the physicochemical composition of OMW is extremely variable as it depends on several factors such as the extraction process, edaphoclimatic, and cultivation parameters, as well as the type, quality, and maturity of the olives [5–7]. OMW typically exhibit strong odor nuisance, acid pH, intensive violet-dark color, considerable saline toxicity reflected by high electroconductivity (EC) values, and very heavy organic pollutants load [5]. Additional difficulties such as small size and geographical dispersion of olive oil mills as well as seasonality of olive oil production are faced in the management of these agroindustry effluents.

In this research work, the treatment of the effluents generated by olive mills working with the two-phase olive oil extraction process (OMW-2) by TiO_2_/UV light photocatalysis is addressed. The aforementioned set of features highlight TiO_2_/UV light photocatalysis as a potential AOP from a technical and economical point of view if compared with other AOPs for the treatment of industrial effluents presenting high organic matter (COD) pollutants load [8–20]. However, the main handicap in applying photocatalytic processes for the reclamation of industrial and agrofood effluents relies in the cost of the catalyst. In this sense, the difficulty in recovering the catalyst poses the main technical-economical drawback. To solve this problem, a novel photocatalyst with ferromagnetic properties for ease of recovery and high effectiveness in the treatment of OMW-2 was developed. Also, the evolution of the organic matter (COD) and degradation rates were determined.

## 2. Experimental

### 2.1. Feedstocks: Olive Oil Washing Wastewater (OMW) and Mixture of Olives and Olive Oil Washing Wastewater (MOMW)

Samples of OWW from the washing machines and OMW at the outlet of the vertical centrifuges were taken from an olive oil mill located in Jaén (Spain) operating with the modern two-phase olive oil extraction procedure [5]. Two different raw feedstocks were used for the present investigation: on one hand, OMW and on the other, a 1 : 1 (v/v) mixture of olives and olive oil washing wastewater (MOMW). The physicochemical characteristics of both raw feedstocks are reported in Table 1.

As it can be noted, the organic load in the effluent from the vertical centrifugation (OMW) is much higher than that from the washing of the olives (OWW) and MOMW, and the same occurs for the concentration of phenolic compounds (Table 1). This is explained on the basis that organic pollutants, and particularly phenolic compounds, are transferred from the oil phase to the water (hydrophilic phase) during the vertical centrifugation, whereas during the olives washing process, the level of organic contamination attained in the water (OWW) is much lower and phenolic species are negligible, only measured in case of fruit rupture in the recollection of the fruit or during the washing procedure [6]. The organic load in OWW stands normally below the limits for discharge on superficial suitable terrains. However, concentration values may exceed the established standards (Guadalquivir Hydrographical Confederation, 2006: COD < 1000 mg O_2_ L^−1^) depending mainly on the water flowrate employed in the olives washing machines during the fruit cleaning procedure.

### 2.2. Pretreatment of the Raw OMW and MOMW Effluents

In first place, both raw feedstocks were subjected to gridding (cut-size equal to 300 *μ*m) with the primary objective of removing coarse particles. After this, a pretreatment procedure based on pH-temperature flocculation, studied in previous works [21], was applied to the OMW and MOMW effluents. In this study, pH-T flocculation process optimization was checked again at lab scale on the target feedstocks and finally conducted on a pilot scale.

Laboratory scale experiments were first carried out in order to find the best pH and temperature conditions for the flocculation process. OMW and MOMW samples (200 mL) were poured in beakers (0.5 L) fitted with magnetic stirring. Experiments at different pH values (ranging from 2 up to 7) and several temperature values (15, 25, and 50°C) were performed. HNO_3_ (70% w/w) and NaOH (1 N) were used to reduce or increase the pH values of the feedstock, respectively. For all experiments, the same procedure was adopted: a short, initial high stirring rate mixing (90 s, 1000 rpm) followed by slow stirring for a longer amount of time (20 min, 320 rpm). The initial strong mixing stage promotes uniform dispersion of the flocculant and particles collisions, whereas the following weak mixing ensures ideal conditions for the movement of the flocks in suspension, without destroying them. After that mixing was completely stopped, the sample was left to settle for 24 hours, and the mud was extracted from the bottom of the reactor and finally dried in order to calculate the sludge fraction and the fraction of clarified water (%v/v). Additionally, total suspended solids (TSS) removal efficiency was measured in the clarified supernatant at the end of each experiment.

Once optimized, the pH-T flocculation process was scaled up and conducted in a stirred batch reactor (20 L) provided with a turbine impeller stirrer and the achieved reduction of total phenols concentration (TPh), chemical oxygen demand (COD), and total suspended solids (TSS) were finally measured.

### 2.3. Lab-Made Production of the Ferromagnetic-Core Nanocatalyst

The possibilities of application of TiO_2_ are being investigated since the early 1970s after a pioneering work by Fujishima and Honda [22] and is nowadays a well-known and commercially used photocatalyst [23]. Nanocrystalline TiO_2_ immobilized on supporting materials such as glass, sand, or zeolite can improve the separation efficiency. Magnetic separation provides a very convenient approach for removing and recycling magnetic particles (such as magnetite, ferrite, and barium ferrite) by applying external magnetic fields. The incorporation of magnetic components into TiO_2_ nanoparticle-based catalysts may, therefore, enhance the separation and recovery of nanosized TiO_2_ [24]. Very recently, a large-scale synthesis of discrete and uniformly sized super paramagnetic Fe_3_O_4_/SiO_2_ was developed [25]. Reaction time, tetraethyl-orthosilicate (TEOS)/Fe_3_O_4_ ratio, and hydrophilic Fe_3_O_4_ seeds concentration were found to be very important parameters in the control of silica shell thickness from 12.5 nm to 45 nm [26].

However, currently there is little literature on the synthesis of Fe_3_O_4_/SiO_2_/TiO_2_ core-shell nanoparticles and their photocatalytic properties. Gad-Allah et al. [27] reported the preparation of Fe_3_O_4_/SiO_2_/TiO_2_ nanocomposites. However, Fe_3_O_4_/SiO_2_/TiO_2_ core-shell nanoparticles were in the form of patches and not discrete nanoparticles; thus, these nanoparticles exhibited reduction of their surface area and photocatalytic properties. Abramson et al. [28] produced core-shell-shell Fe_3_O_4_/SiO_2_/TiO_2_ nanoparticles of few tens nanometers by successively coating onto the magnetic nanoparticles a SiO_2_ layer and a TiO_2_ layer, using sol-gel methods.

**Figure 2:**
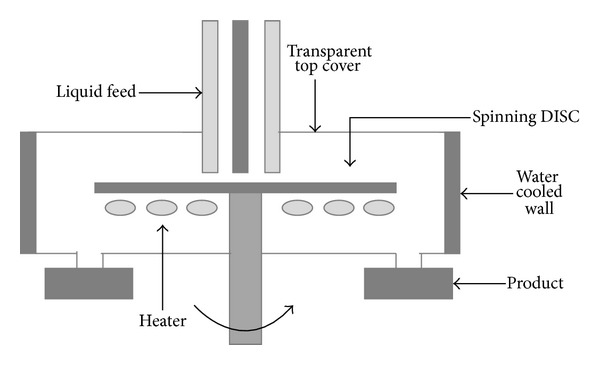
Scheme of the adopted rotating disk reactor [21, 29].

In this work, the production process of the photocatalyst was performed in three subsequent steps. Firstly, magnetite was produced by using a spinning disk reactor (SDR). This technology allows obtaining nanomaterial by chemical precipitation or sol-gel processes continuously. Two reactants were used: on the one side, an aqueous solution of FeCl_3_, HCl, and Na_2_SO_3_ and on the other side, an aqueous solution of NH_4_OH. As pointed out previously by De Caprariis et al. [29], the location of the feed points over the disk influences the precipitation outcome. In this case, the first reactant was fed at the center of the disk, whereas the second one was injected at 2 cm of distance. This permits producing magnetite with a modal particle size of 30 nm. A scheme of the adopted spinning disk reactor is shown in Figure 2.

The second step consists of a coating of silica, performed by adding the dried magnetite particles to a TEOS-ethanol-NH_3_ solution. The coated particles were then recovered back by magnets, gently dried at 80°C, and calcinated at 450°C.

Finally, the TiO_2_ coating was performed by pouring the silica-coated particles in a titanium tetraisopropoxide-ethanol solution and adding H_2_O_2_ dropwise to the solution under strong mixing conditions. Again, the recovered particles were dried gently at 80°C and a final calcination at 450°C was performed.

### 2.4. Characterization of the Lab-Made Photocatalyst

Particle size distribution (PSD) analysis was performed with a dynamic light scattering device (Plus90 nanosizer) supplied by Brookhaven. Determination of the presence of silicon and titanium atoms was conducted by means of an energy-dispersive X-ray diffraction (EDX) analysis. Transmission electron microscopy (TEM) was carried out with an Auriga Zeiss instrument to observe the overall morphology of the nanoparticles.

### 2.5. TiO_2_/UV Photocatalytic Degradation of OMW and OWMW

TiO_2_/UV photocatalysis process was firstly optimized at lab scale on 200 mL samples of the supernatant of OMW and OWMW previously pretreated by pH-T flocculation. Lab experiments were conducted in 0.5 L beakers with magnetic stirring at ambient temperature (20 ± 0.5°C) and medium agitation speed (500 rpm) during 2–4 hours under irradiation of an UV lamp (nominal power 45 W, wavelength 365 nm). The performance of the lab-made ferromagnetic-core photocatalytic nanoparticles was contrasted with that of commercial TiO_2_ P-25 catalyst provided by Degussa for comparison purposes. Different catalysts dosages (0.5, 1, 3, and 9 g L^−1^ for both catalysts, plus 20 g L^−1^ for the commercial Degussa P-25) were tested and reduction of the COD values was followed during the course of the experiments. Laboratory scale experiments aimed to find the best TiO_2_ catalyst and its optimal initial dosage.

Once lab-scale optimization was accomplished, the photocatalysis process was carried out in an agitated batch reactor (8 L), provided with an UV lamp on top and a turbine impeller stirrer. Achieved reduction of COD, total phenols (TPh) concentration, and total suspended solids (TSS) were checked at the end of the pilot scale process.

### 2.6. Analytical Methods

Chemical oxygen demand (COD), total phenols (TPh), total suspended solids (TSS), electroconductivity (EC), and pH were measured following standard methods (Greenberg, 1992). All analytical methods were applied in triplicate with analytical-grade reagents, including 70% (w/w) HNO_3_, 98% (w/w) NaOH, 98% (w/w) Na_2_SO_3_, 30% (w/w) NH_4_OH, 37% (w/w) HCl, and 30% (w/w) FeCl_3_, supplied by Panreac.

## 3. Results and Discussion

### 3.1. Pretreatment of OMW by pH-T Flocculation

**Figure 3:**
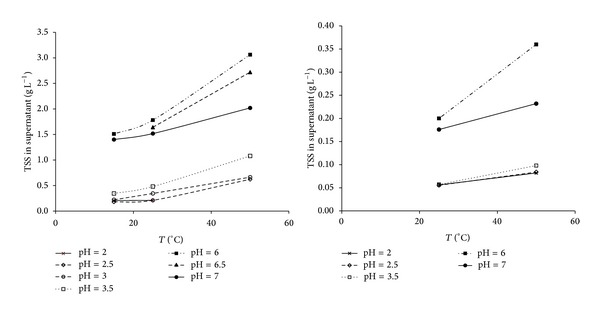
Lab-scale results of pH-T flocculation process on OMW (left caption) and MOMW (right capt.).

Figure 3 shows the optimal operating conditions found at lab scale for the pH-T flocculation process, taking TSS reduction in supernatant as key parameter, suggested working at temperatures of 25 ± 0.5°C and pH values equal to 2.5 ± 0.25 (by adding 0.55 ± 0.05% v/v 70% HNO_3_). OMW is considered to be closely related to humic compounds because it is dark colored, contains phenols, and shares some of the properties of humic substances. Jin et al. [30] studies on particle size distribution (PSD) of humic acid by dynamic light scattering confirmed higher hydrodynamic diameters at lower temperature values, predominantly in form of submicron aggregates and a small fraction of supramicron aggregates, due to minor solubility of the organic matter. Furthermore, at low pH values (pH < 4), these macromolecules are protonated and thus neutrally charged. The absence of electrostatic repulsion among the humic macromolecules thus enhances molecular aggregation. Similar trends with regard to natural organic matter (NOM) macromolecules were observed by Hong and Elimelech [31].

Upon those operating conditions, the final reduction values achieved at the end of the pilot scale pH-T flocculation process were equal to 72.5% TSS, 12.6% COD, and 5.0% TPh, with a recovery of up to 87.5% v/v of the clarified water which means a resultant 12.5% v/v of sludge. In a similar way, for the MOMW effluent, these results were equal to 93.4% TSS, 14.3% COD, and 5.2% TPh reduction efficiencies. In this case, a final recovery of up to 90.2% v/v of the clarified supernatant was accomplished, that is leading to a resultant 9.8% v/v of sludge. The pH-T coagulation-flocculation process seems to be a cost-effective pretreatment for the removal of the TSS concentration from OMW and MOMW, particularly if compared to current coagulation-flocculation processes with commercial flocculants, which may lead in our experience to lower efficiency values at higher costs.

### 3.2. Characterization of the Lab-Made Photocatalyst

**Figure 4:**
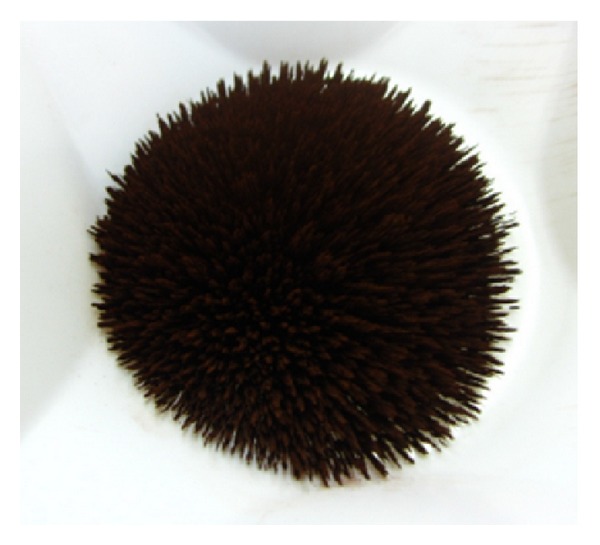
Photography of the ferromagnetic photocatalytic nanoparticles.

**Figure 5:**
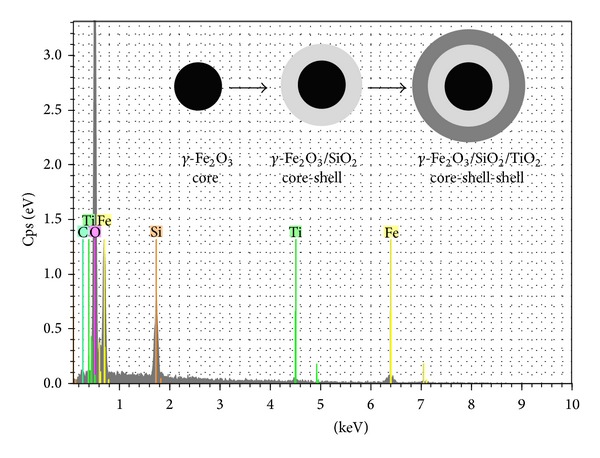
Energy-dispersive X-ray diffraction spectrum (EDX) of the ferromagnetic nanoparticles.

**Figure 6:**
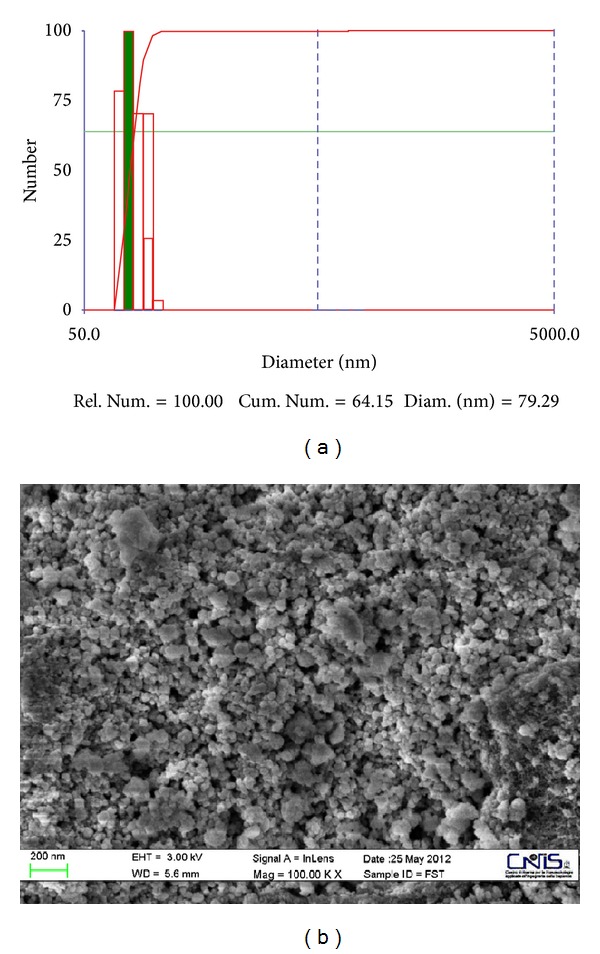
Particle size distribution measured by the nanosizer (a) and TEM photograph (b) of the final ferromagnetic catalyst particles.

The lab-made nanocatalyst (Figure 4) is internally composed of a ferromagnetic core (*γ*-Fe_2_O_3_, modal particle size of 30 nm) on which two subsequent layers of silica and titania are attached (*γ* Fe_2_O_3_/SiO_2_/TiO_2_). Energy-dispersive X-ray diffraction spectrum (EDX) of the ferromagnetic nanoparticles is given in Figure 5, whereas transmission electron microscopy (TEM) microphotographs and particle size distribution (PSD) analysis of the obtained ferromagnetic-core nanoparticles are shown in Figure 6. TEM image of the nanoparticles shows regular overall morphology. The final mean particle size of the nanopowder was equal to 79 nm with very uniform, homogeneous, and pure anatase TiO_2_ phase (100%) with some traces of brookite. EDX analysis, performed to determine the elemental composition of the obtained composite nanoparticles, shows that the obtained nanopowder consisted of Fe, Si, and Ti, verifying the presence of both silica and TiO_2_ layers.

### 3.3. Photocatalytic Degradation of OMW

**Figure 7:**
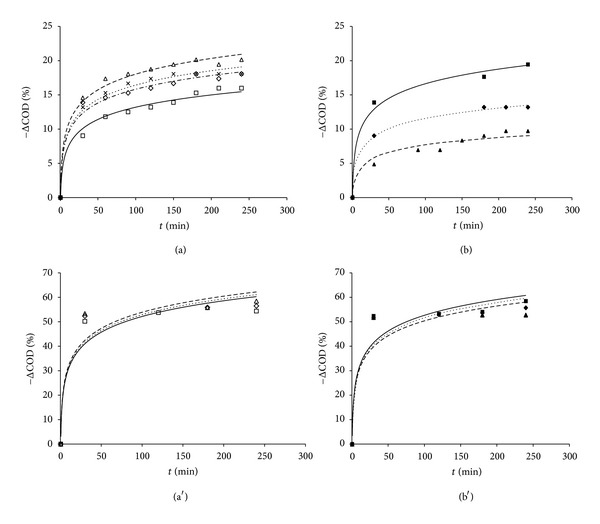
Results withdrawn from UV/TiO_2_ photocatalysis experiments on lab scale with OMW: commercial Degussa P-25 (a): □ = 1 g/L, *◊* = 3 g/L, Δ = 9 g/L, x = 20 g/L; lab-made ferromagnetic-core nanopowder (b): ■ = 1 g/L, ♦ = 3 g/L, ▲ = 9 g/L; MOMW: Degussa P-25 (a′): □ = 1 g/L, *◊* = 3 g/L, Δ = 9 g/L; lab-made ferromagnetic-core nanopowder (b′): ■ = 0.5 g/L, ♦ = 1 g/L, ▲ = 3 g/L.

The evolution of the organic matter (COD) removal from OMW during the lab-scale UV/TiO_2_ process with both catalysts and feedstocks is reported in Figure 7. A semilogarithmic fitting curve was applied for interpolating the COD reduction during operation time as follows:

(5) $$\begin{array}{c} \Delta \text{COD}=B\cdot \ln\left(t\right)+\Delta \text{COD}\left(0.5\right), \end{array}$$

where *B* (g L^−1 ^h^−1^) indicates the rate of degradation of the organic matter during the photocatalysis experiments and ΔCOD (0.5) (g L^−1^) makes reference to the initial abatement of organic pollutants. Overall results of the lab-scale UV/TiO_2_ tests are reported in Table 2.

The laboratory-made ferromagnetic-core photocatalyst was found to provide slightly higher COD removal than the commercial one for both feedstocks upon lower initial catalyst dosages, equal to 1 g L^−1^ and 0.5 g L^−1^ for the treatment of OMW and MOMW, respectively. This is supported by the 100% anatase TiO_2_ phase of the lab-made ferromagnetic catalyst. Among the three crystalline phases of TiO_2_, called anatase, rutile, and brookite, the former is the most active under UV irradiation, strongly dependent on the particle size and habit too [32]. The sol-gel process performed in this work for the fabrication of the ferromagnetic core catalyst leads to the formation of homogeneous, pure, and very uniform particles (Figure 6(b)) [29]. Mixed-phase submicron particles such as commercial Degussa P-25, consisting of 70% anatase and 30% rutile, seem to provide less effective catalytic action.

Moreover, significantly higher organic matter removal efficiency was observed for the MOMW effluent than that for OMW upon the optimal catalyst dosage, 58.4% for the former in contrast with 21.4% for the latter (Table 2). Also, adding large quantities of titania to the wastewater leads to poorer results. Both effects are narrowly connected to hindrance to deep penetration of UV light as a result of the opacity of the solutions, due to the major organic pollutants load or to the excessive nanocatalyst concentration, respectively, which in turn impedes efficient activation of the catalyst. A guided focus on Figure 7 permits to notice that no significant increase of the COD removal efficiencies was observed beyond a residence time (*τ*) of 3 hours for OMW, whereas lower residence time was needed in the case of MOMW, *τ* = 2 h.

Finally, UV/TiO_2_ photocatalysis process was scaled-up. The physicochemical composition of both OMW and MOMW exiting each pilot scale treatment stage is given in Table 3. By adopting the optimal conditions at pilot scale, 23.4% COD, 19.2% TPh, and 28.1% TSS were efficiently removed at the end of UV/TiO_2_ process for OMW, whereas 58.3% COD, 27.5% TPh, and 25.0% TSS for MOMW.

Again at lab scale, not only sensibly higher COD removal efficiency was noted (23.4% versus 58.3%) but also phenolic compounds (TPh) abatement (19.2% versus 27.5%) for MOMW if compared with OMW, upon the optimized operating conditions, due to more efficient activation of the nanocatalyst owed to deeper penetration of UV light in less opaque medium.

The novel prepared lab-made titania ferromagnetic-core photocatalyst (*γ*-Fe_2_O_3_/SiO_2_/TiO_2_) was developed with the goal of being able to be whether recovered back from the wastewater stream by a magnetic trap and reused or even fixed to the photocatalysis reactor, giving a solution to the problem of the recovery of the catalyst and thus considerably enhancing the cost-effectiveness of the olive mill effluents reclamation process.

Furthermore, the overall efficiency of the process by treating the mixture of the OMW stream mixed with the OWW effluent (MOMW) triggers up to 91.0% of COD removal, leading to final COD values in the treated water below 1.5 g L^−1^, in contrast with the barely 33.2% COD removal efficiency achieved for the treatment of the OMW separately.

## 4. Conclusions

According to the results obtained in this investigation, the photocatalytic degradation process is an alternative with high possibility to be used in the treatment of OMW from two-phase continuous centrifugation process. The easy recovery and reuse of photocatalyst minimizes the operating costs of the treatment process. The novel photocatalyst used in this work offers good results in comparison with other commercial catalysts as Degussa P-25. The photodegradation process implies high reduction of the percentages of COD, total phenols, and total suspended solids. At pilot scale, 23.4% COD_removal_, 19.2% total phenols_removal_, and 28.1% total suspended solids_removal_ were registered at the end of the UV/TiO_2_ process for OMW upon a residence time (*τ*) equal to 3 hours, whereas 58.3% COD_removal_, 27.5% total phenols_removal_, and 25.0% total suspended solids_removal_ for MOMW upon *τ* = 2 h. Also, before the UV/TiO_2_ reaction a pH-T flocculation operation as a pretreatment was realized. The overall efficiency of the treatment process for MOMW was up to 91% of COD_removal_, in contrast with 33.2% of COD_removal_ for OMW.

## Figures and Tables

**Table 1:** Raw OMW and MOMW physicochemical composition.

Parameters	OMW	MOMW
pH	4.9–5.1	5.9–6.3
Electric conductivity, mS cm^−1^	1.76–1.84	1.42–1.54
Total suspended solids, g L^−1^	3.1–5.8	6.1–6.9
COD, g L^−1^	16.4–16.6	4.1–4.2
Total phenols, g L^−1^	0.181–0.184	0.082–0.087

**Table 2:** COD reduction in OMW and MOMW after lab-scale UV/TiO_2_ photocatalysis.

Raw effluent	Catalyst type	Catalyst dosage g L^−1^	COD_final_ g L^−1^	−ΔCOD_4h_ %	ΔCOD (0.5) g L^−1^	*B * g L^−1^ h^−1^
OMW (COD_initial_ 14.5 g/L)	Degussa P-25	1	12.1	16.5	1.3	2.6
		3	11.8	18.6	2.5	2.9
		9	11.5	20.7	2.8	3.3
		20	11.8	18.6	2.7	3.0
	Ferromag. TiO_2_	1	11.4	21.4	2.5	3.2
		3	12.5	13.3	1.6	2.2
		9	13.1	10.3	0.6	1.6

MOMW (COD_initial_ 3.6 g/L)	Degussa P-25	1	1.7	54.3	10.1	9.2
		3	1.6	56.5	10.0	9.3
		9	1.5	57.0	10.1	9.5
	Ferromag. TiO_2_	0.5	1.5	58.4	9.8	9.3
		1	1.6	55.7	10.0	9.1
		3	1.7	52.7	9.9	8.7

**Table 3:** Physicochemical composition of OMW and MOMW after flocculation (F) and photocatalytic (PC) degradation on pilot scale.

Feedstock	OMW-F	OMW-F and PC	MOMW-F	MOMW-F and PC
pH	2.5	2.9	2.5	3.1
EC, mS cm^−1^	1.7	1.8	1.5	1.5
TSS, g L^−1^	1.6	1.2	0.4	0.3
COD, g L^−1^	14.5	11.1	3.6	1.5
TPh, mg L^−1^	172	139	82.5	59.8
